# Implementation of a Whole‐School Relationship and Sexuality Education Project in Western Australian Schools: Evaluation Data From a Multiple, Embedded Case Study

**DOI:** 10.1002/hpja.70138

**Published:** 2025-12-03

**Authors:** Sharyn Burns, Roisin Glasgow‐Collins, Hanna Saltis, Jacqueline Hendriks

**Affiliations:** ^1^ Collaboration for Evidence, Research and Impact in Public Health Curtin University Perth Australia; ^2^ Curtin School of Population Health Curtin University Perth Australia

**Keywords:** embedded case study, health promoting schools, implementation, relationships and sexuality education, whole‐school

## Abstract

**Issue Addressed:**

School‐based relationships and sexuality education (RSE) offers broad social, emotional and physical health benefits. However, many teachers feel unprepared to deliver comprehensive, contemporary RSE. To address this, the Curtin RSE Project supported schools in implementing whole‐school RSE using a Health Promoting Schools framework, through a supported case study intervention.

**Methods:**

A multiple, embedded case study design was employed to evaluate implementation in participating case study schools. Of the four schools recruited, data were collected from one primary and one secondary school. This paper reports on student surveys (Grades 6 and 7–12) and focus group discussions and interviews with teachers, parents and students (Grades 9–12). Descriptive statistics were generated for the surveys and reflexive thematic analysis conducted for qualitative data.

**Results:**

Students found RSE relevant, though some reported discomfort during sessions. The breadth of RSE topics in the primary school increased post‐intervention. Four themes were reflexively identified to understand RSE implementation: *RSE is systematically not prioritised; Approaches to age‐appropriate education; Schools are appropriate environments; and A whole‐school approach for success.*

**Conclusions:**

Support from school leadership was essential for proper implementation of whole‐school RSE, supported by passionate and well‐supported school staff. Whole‐school approaches ensured consistent messages within the school and home environment.

**So What?:**

There is a pressing need to embed RSE in pre‐service teacher education and to provide coordinated, ongoing professional learning for in‐service teachers. Provision of technical support for schools facilitates implementation. These strategies will ensure delivery of contemporary, high‐quality RSE and support sustainable whole‐school implementation.

## 1 Introduction

School‐based relationships and sexuality education (RSE) has demonstrated positive outcomes for adolescent social and emotional development [1, 2, 3]; reduced the frequency of unprotected sexual activity and unintended pregnancies and delayed the onset of first sexual debut [1, 3, 4, 5]. International guidelines for school‐based RSE [3] suggest an array of topics such as gender diversity [6, 7], respectful relationships [8, 9], digital literacy [10] and pornography [11] be included. This has been widely emphasised and supported by students [9, 11], educators [7, 11] and parents [12, 13, 14].

Despite this guidance, in the Australian context, school‐based RSE provision varies. Guidance specific to RSE within the Australian curriculum is purposively vague to allow schools the flexibility to adapt teaching and learning content to their school context. This provides an opportunity to respond to cultural diversity, community expectations and students' needs [15]. Furthermore, while an Australian curriculum exists [16] some states and territories provide modified versions [17, 18, 19]. Inconsistent provision is also impacted by a lack of professional learning in RSE at both the pre‐service [20] and in‐service level [21, 22]; and that Health, as a learning area, is not always closely regulated or moderated within schools [21] or afforded the same priority as other learning areas [23]. While some Australian studies highlight dissatisfaction with current RSE provision amongst students [24, 25], educators [20, 26] and parents [27], schools are seen as important settings for RSE [12].

Comprehensive RSE recognises socioecological influences [28] and the complex interactions between individuals, interpersonal, organisational, community and societal factors. This makes whole‐school approaches suitable [29]. The Health Promoting Schools framework advocates for a whole‐school approach, encompassing eight key areas: government policies and resources, school policies and resources, school governance and leadership, school and community partnerships, school curriculum, school social–emotional environment, school physical environment and school health services [30]. The framework promotes practices, policies and procedures to reinforce classroom activities and to embed culture throughout the school, including in communication with key stakeholders and strategic directions [30, 31]. This framework has utility for all stages of project implementation, including needs assessment, design, implementation and evaluation.

While Australia has a long history of advocating for whole‐school approaches, focus has traditionally been on health topics such as nutrition and physical activity [32, 33], bullying [34, 35], alcohol and other drugs [36, 37] and road safety [37, 38]. Reported evaluation of whole‐school implementation of RSE is minimal. One Australian programme cited the ad‐hoc nature of RSE delivery, mostly within a classroom setting, varying levels of support from the broader school and parent community, concerns regarding topics covered and an overcrowded curriculum [23]. These barriers, in addition to a lack of experience and familiarity with contemporary RSE content, may result in teachers feeling ill‐equipped and unprepared to deliver RSE content [21, 23].

Since 2014, the Curtin University RSE Project has been funded by the Western Australian Health Department to deliver professional learning to pre‐service and in‐service school staff, across kindergarten to grade 12 (K‐12), to strengthen workforce capacity to provide comprehensive RSE in Western Australian schools (https://rse.project.org.au). An undergraduate unit for pre‐service teachers [39] and 2‐day interactive workshops for pre‐service school staff [40], have generated positive process and short‐term impact evaluation data. These strategies increased confidence and competence to deliver RSE and the development of positive attitudes towards RSE provision [39, 40]. While the short‐term impact of professional development for preservice and in‐service teachers is encouraging there is limited evidence of the effectiveness of the implementation of whole‐school RSE.

A case study approach was employed to answer the research question ‘What supports whole‐school RSE implementation?’ This paper reports findings from the cross‐sectional survey conducted with students and qualitative data collected through focus group discussions (FGDs) and interviews with parents, teachers and students.

## 2 Methodology

A multiple, embedded case study design [41] was employed to measure the implementation of whole‐school RSE strategies; to explore the perceptions of students, teachers and parents towards RSE; and to identify barriers and enablers to comprehensive school‐based RSE [42]. A multiple case study design allows for the participation of primary (K‐Grade 6) and secondary (Grades 7–12), geographic and school size variations. An embedded case study design allows for the analysis of various strategies at different levels within the broader health‐promoting school. This allowed schools to focus on pedagogical and locally appropriate strategies relevant to their school community [30, 41].

### 2.1 Case Study Sites

Schools were invited to participate as a case study school via an expression of interest to parties registered on the RSE Project database. Case study sites were purposively selected based on their capacity to commit to the project for an initial 2‐year period and approval from their school leadership team. Four schools were included as case study schools comprising a primary (School 1), secondary (School 2), remote district high (K‐Grade 10) (School 3) and secondary education support centre (School 4).

Three schools were metropolitan (Schools 1, 2 and 4), and one was in a remote area in Western Australia. All schools were government co‐educational secular schools. Two schools that commenced working as case study schools opted out (Schools 3 and 4). Both schools were unable to establish an ongoing committee. The education support centre had a very committed staff member, who took long service leave and commitment was not maintained by other staff in their absence. The remote school experienced severe flooding resulting in the closure of the school for a period, followed by the COVID‐19 pandemic. The RSE team travelled to this site twice early in the project; however due to pandemic travel restrictions, they were unable to conduct follow‐up visits. While online support was provided this was not sufficient to maintain progress. School demographics are described in Table 1.

**TABLE 1:** Demographics and implementation of whole‐school RSE by school.

	School type	ICSEA^a^	Student/FTE staff	Strategies implemented	Evaluation data reported
School 1	Primary (Grades K‐6)	> 1000	Students: *N* = 450 Staff: *N* = 450	3 staff attended 2‐day workshop 1 staff completed additional university degree (GD‐SXLGY) School climate and school curriculum audit of current RSE provision Establishment of RSE Committee (*n* = 4–6 school staff, mix of genders). 1–2 meetings each school term. 90‐min workshop for all school staff (baseline) Review of all applicable school policies Review of health programme scope and sequence Curriculum connections across various learning areas identified Session for Year 6 parents and children related to sexuality, sexual health and online safety Targeted focus on how gender is addressed within the school across Days of Celebration: White Ribbon Day, Harmony Day, NAIDOC Week, Wear it Purple Day (staff only). Events included guest speakers, fundraising or awareness raising activities, whole‐school activities or classroom lessons connected to the topic. Emphasis on obtaining student feedback regarding all RSE‐related classroom lessons and school initiatives Regular communication with parents and families regarding RSE‐related classroom lessons or school initiatives (e.g., emails to parents, school newsletters, announcement at assemblies, social media posts) School‐directed evaluation of all activities (i.e., obtaining feedback from staff, students and families)	Surveys (baseline and post‐intervention) Grade 6 students pre‐intervention survey (*n* = 44) Grade 6 students post‐intervention survey (*n* = 47) Interviews and focus group discussions (post‐intervention): – Teacher one‐on‐one interviews (*n* = 1) – Parent interviews (*n* = 3) – One teacher FGD (*n* = 3)
School 2	Secondary (Grades 7–12)	< 1000	Students: *N* = 1220 Staff: *N* = 80.1	2 staff attended 2‐day workshop School climate and school curriculum Audit of current RSE provision Establishment of an RSE working party/committee (*n* = 20+ school staff and community organisations, mix of genders). 1–2 meetings each school term. 90‐min workshop for all school staff (baseline) Review of all applicable school policies Half‐day workshop for all school staff (post‐intervention) 2‐h workshop for HPE Team focused on teaching and learning pedagogy and resources Review of health programme scope and sequence Curriculum connections across various learning areas identified RSE lessons modelled for all year groups (7–12) across the 3 years RSE guest speakers secured for presentations to all year groups (7–12) Targeted focus on respectful relationships Targeted focus on improved connection with local police service through a Brazilian Jui‐Jitsu programme with a purposeful selection of high‐risk students Targeted focus on RSE that is culturally safe for Aboriginal students (2 staff members trained in Sexual Health Quarter's Modiji Program) Purposeful acknowledgement or particular Days of Celebration: White Ribbon Day, Harmony Day, NAIDOC Week, Wear it Purple Day, IDAHOBIT (International Day Against Homophobia, Biphobia and Transphobia). Events included guest speakers, fundraising or awareness raising activities, whole‐school activities or classroom lessons connected to the topic. Emphasis on obtaining feedback from all students regarding all RSE‐related classroom lessons and school initiatives. Purposeful engagement with the Student Leadership Group. Regular communication with parents and families regarding RSE‐related classroom lessons or school initiatives (e.g., emails to parents, school newsletters, announcement at assemblies, social media posts) School‐directed evaluation of all activities (i.e., obtaining feedback from staff, students and families)	Surveys (baseline): – Grade 7–12 pre‐intervention survey (*n* = 545) Interviews and focus group discussions (post‐intervention): – Teacher on‐on‐one interviews (*n* = 2) – One teacher FGD (*n* = 4) – One student FGD (Leadership Group Grades 9–12) (*n* = 5)
School 3	Regional (Grades K‐12)	< 1000	Students: *N* = 130 Staff: *N* = 16.4	All staff attended 2‐day workshop School climate and school curriculum Audit of current RSE provision Review of health programme scope and sequence Curriculum connections across various learning areas identified Targeted focus on protective behaviours Targeted focus on RSE that is culturally safe for Aboriginal students (1 staff member trained in Mooditj Program, previous community members who had also been trained in Mooditj were also identified)	School lost to follow‐up for multiple reasons: – Unable to establish a working party – Severe flooding in the area closed the school temporarily and this was followed closely by the COVID‐19 pandemic – RSE Project Team only able to travel to the site twice early in the project and were unable to conduct follow‐up visits, support via email/phone/video conference was not sufficient
School 4	Education support (Grades 7–12)	> 1000	Students: *N* = 50 Staff: *N* = 7.6	2 staff attended 2‐day workshop School Climate and School Curriculum Audit of current RSE provision Review of health programme scope and sequence	School lost to follow‐up as they were unable to establish a working party. The most committed staff member took long service leave and commitment to the project was not maintained.

Abbreviation: FTE: full‐time employee.

^a^ 1000 represents average ICSEA (index of community‐socio‐educational advantage); < 1000 lower ICSEA; > higher ICSEA.

### 2.2 Intervention

Case study schools formed an RSE‐focused Committee. Committee members varied; however, all included various school staff and community members. School staff from Schools 1 and 2 included senior administration and teachers. Committees included a mix of genders. The RSE committee for School 2, a large secondary school, also comprised community organisations (Table 1). RSE team staff actively participated as members of each committee, providing technical guidance and support.

Schools received direct support to plan and implement RSE strategies specific to their school community. RSE Project staff supported the school committee to complete a school climate audit and school curriculum audit [42] enabling schools to select initiatives based on their school community needs.

As part of the audit process all schools identified the need for professional development. Two to three staff members from each case study school were invited to participate in a 2‐day professional development workshop facilitated by the RSE team. For the regional school, the RSE team facilitated an onsite workshop for all school staff. The professional learning strategy outlines key evidence‐based principles that underpin RSE provision and facilitators demonstrate a range of interactive and engaging teaching and learning strategies suitable for primary and secondary school contexts. The training includes curriculum‐based strategies and strategies to promote whole‐school RSE [40].

Across all four sites RSE team staff delivered an initial interactive workshop to all school staff outlining the contemporary scope of RSE, how a whole‐school approach works and how all staff have a role to play. Team staff also reviewed current Health programmes and worked with school staff on scope and sequence adjustments to ensure best‐practice RSE. The initial 2‐year programme was extended to 3 years for the two continuing schools at their request. Specific intervention strategies are described in Table 1.

### 2.3 Evaluation Measures

Several case study evaluation measures were employed [42]. This paper describes quantitative and qualitative data collected from students, teachers and parents from two of the four case study schools (School 1: primary; School 2: secondary). Evaluation measures relevant to this paper are outlined in Table 1. Other findings will be reported elsewhere.

#### 2.3.1 Cross‐Sectional Surveys

Primary (Grade 6, 10–12 years) and secondary (Grades 7–12, 11–18 years) students participated in baseline surveys. Grade 6 students participated in post‐intervention surveys. All students were invited to participate; surveys were conducted online during class time and completion time was approximately 15–20 min.

Students were asked to identify their most trusted sources for RSE information and advice, which subjects covered RSE, the year levels they had received RSE, topics covered, facilitation methods, the relevance of content and who facilitated these lessons [42]. Other questions included students' level of comfort with RSE topics, which was measured through self‐rating how they felt during RSE lessons (embarrassed, annoyed, uncomfortable) on a three‐point Likert scale (always, sometimes, never) [42]. Questions had been validated previously with Australian students (Grades 7–12) [42, 43].

#### 2.3.2 FGDs and Interviews

Semi‐structured FGDs were the primary form of data collection; interviews were offered if preferred by the participant. All FGD/interviews were conducted in person at the school during the final year of the project, audio recorded and transcribed verbatim. Students from the School Leadership Group (School 2; Grades 9–12) were invited to participate in the student FGD. FGDs ran for approximately 60 min and interviews for 30–45 min. Interview guides were modified for each participant group (teachers, students, parents). Participants were invited to reflect on their experience of being a case study school for the RSE Project, with discussion focused on the implementation of whole‐school RSE.

### 2.4 Data Analysis

Survey data were analysed using IBM SPSS Statistics (V29). Descriptive analysis was employed. *χ* ^2^ tests were used to examine associations between RSE relevance and feelings and gender and grade. Significance was assumed at *p* < 0.05.

Reflective thematic analysis was employed to analyse qualitative data. This approach recognises the active role of identifying and interpreting themes [44]. Recognising existing constructs, a deductive approach was used. The six phases of reflexive thematic analysis were followed. Initially data were read and re‐read to ensure familiarisation. This process determined credibility and maintained dependability [45]. Phase 2 involved the initial coding of the data and the creation of meaningful descriptions. During Phase 3, to enhance confirmability [45], the research team began to generate initial themes guided by the data, the research questions and the team's knowledge and insight. Phase 4 involved the team working together to develop and review themes, considering relationships between and within themes. Phase 5 involved refining, defining and naming the themes before the final phase where the write‐up process ensured the data were finessed [44]. Data were managed and analysed using NVivo (V.15) software.

The research team acknowledges our interpretation of the data may be influenced by our professional backgrounds in health promotion (S.B., J.H.), sexology (J.H., R.G.‐C., H.S.) and education (S.B.). We are a team of experienced practitioners and researchers and early career researchers with a research focus on RSE. Our experience and training are likely to inform our perspectives around comprehensive RSE, equity, inclusion and sociological influences. Our genders (female and non‐binary) may also influence our data interpretation. Reflexive thematic analysis ensures we recognise our interpretations are co‐constructed and shaped by our disciplines and experiences.

### 2.5 Ethical Considerations

Ethical approval was obtained from Curtin University Human Research Ethics Committee (HR91/2014) and site approval from the Department of Education Western Australia (D18/0057006).

School principals provided consent for the school to participate. All participants were provided information sheets describing their participation in the study. Information sheets were emailed to teachers and parents and provided in hard copy to students. Teachers and parents provided consent and students provided assent. All participation was voluntary, and students could opt to catch up on homework during data collection if they chose not to participate. No identifying data were collected for surveys; FGD/interview data were de‐identified, data were coded and pseudonyms allocated.

## 3 Results

For School 1, teachers participated in one FGD (*n* = 3) and teachers and parents participated in interviews (*n* = 4). Grade 6 students completed online surveys at baseline (*n* = 44) and post‐intervention (*n* = 47). Two interviews and one FGD (*n* = 4) were conducted with teachers in School 2. Students (*n* = 5) from the school leadership group (Grades 9–12) participated in one FGD. All students from Grades 7 to 12 were invited to participate in surveys at the beginning of the project; 545 completed the survey. Table 2 describes the demographics of survey participants from the two case study schools.

**TABLE 2:** Demographics of student survey participants.

Demographic	School 1 (primary) (pre) (*n* = 42)	School 2 (secondary) (pre) (*n* = 545) (%)	School 1 (primary) (post) (*n* = 47)
Gender			
Male	28 (66.7)	241 (44.5)	21 (44.7)
Female	13 (31)	274 (50.6)	24 (51.1)
Other	—	12 (2.2)	1 (2.1)
Prefer not to answer	1 (2.3)	15 (2.8)	1 (2.1)
Year level			
6	42	—	46
7	—	127 (23.3)	—
8	—	90 (16.5)	—
9	—	119 (21.9)	—
10	—	129 (23.7)	—
11	—	57 (10.5)	—
12	—	22 (4)	—

Four themes were developed from the qualitative data to describe participant's perceptions of RSE, and barriers and enablers to comprehensive school‐based RSE promotion across the two schools (see Table 3). Quantitative and qualitative findings are reported by school.

**TABLE 3:** Key themes describing implementation of whole‐school RSE.

Key theme	Summary
Is RSE a priority?	RSE is not seen as a priority by the education system. Lack of allocated time to allow teachers to cover all areas of a comprehensive RSE programme. Schools are under pressure to deliver RSE among other competing priorities in Australian curriculum. Individual teachers are passionate about delivering evidence‐based RSE
Age‐appropriate education	RSE needs to be delivered in a way which is appropriate for specific age groups. Primary school believed in tailoring all content so deliver all information in age‐appropriate ways. Secondary school focused on excluding content based on maturity.
A whole‐school approach is necessary	RSE should be covered through other subjects, not only HPE. A whole‐school approach involves aspects broader than curriculum, including the school's environment and teacher modelling. A top‐down approach is necessary.
Schools are appropriate environments for RSE delivery	Schools should provide an unbiased environment, where teachings are not influenced by personal beliefs. School based‐RSE enables consistency of message

### 3.1 School 1: Quantitative Findings

Of those who completed the baseline survey (*n* = 42), 51.9% reported having been taught RSE previously. RSE was mostly included in Health (*n* = 20; 47.6%), ‘other’ subjects (*n* = 10; 23.8%) and Science (*n* = 7; 16.7%). Sessions were mostly facilitated by a classroom teacher (*n* = 20; 45.5%), the school nurse (*n* = 9; 20.5%) and an external facilitator (*n* = 9; 20.5%).

Most students reported the RSE they received in school was ‘somewhat relevant’ (*n* = 17; 42.5%) or ‘very relevant’ (*n* = 12; 30%). Females were more likely to provide these responses than males (*χ* ^2^ = 18.477, df = 8, *p* = 0.018) Commonly reported RSE topics that were covered included emotions and bullying (*n* = 31; 70.5%), cyberbullying (*n* = 30; 68.2%) and friendships (*n* = 28; 63.6%). Few students reported having been taught about the male (*n* = 2; 4.5%) and female reproductive systems (*n* = 6; 13.6%), menstruation (*n* = 4; 9.1%) and love and intimacy (*n* = 3; 6.8%) (Table 4).

**TABLE 4:** Topics covered in School 1 at pre‐ and post‐intervention.

Topic	Pre‐intervention		Post‐intervention	
	*N*	%	*N*	%
Bullying	31	70.5	42	89.4
Emotions	31	70.5	45	95.7
Cyber bullying	30	68.2	43	91.5
Friendships/how to be a good friend	28	63.6	43	91.5
How to stay safe online	23	52.3	43	91.5
‘Our bodies are our own’	21	47.7	44	93.6
How to show respect	17	38.6	38	80.9
Early warning signs	13	29.5	35	74.5
Changes that happen during puberty	13	29.5	41	87.2
Relationships	12	27.3	44	93.6
Gender identity	9	20.5	41	87.2
Where babies come from	8	18.2	41	87.2
Female reproductive system	6	13.6	45	95.7
Menstruation	4	9.1	30	63.8
Male reproductive system	2	4.5	44	93.6
Love and intimacy	3	6.8	19	40.4
Something else	3	6.8	2	4.3
We have not covered any of these topics	3	6.8	0	0

Some students reported some level of discomfort during RSE (*n* = 19; 51.4% ‘sometimes’ and *n* = 7; 18.9% ‘always’ felt uncomfortable). Over half ‘never’ felt embarrassed during RSE sessions (*n* = 20; 55.6%) (*n* = 13; 36.1% ‘sometimes’ and *n* = 3; 8.3% ‘always’ embarrassed). There were no significant differences between feeling embarrassed (*χ* ^2^ = 8.051, df = 4, *p* = 0.090), uncomfortable (*χ* ^2^ = 2.685, df = 4, *p* = 0.617) or annoyed (*χ* ^2^ = 2.200, df = 4, *p* = 0.699) and gender.

At post‐intervention (*n* = 47) almost all students reported they had been taught RSE previously (*n* = 45; 95.7%). Lessons were mostly delivered during Health (*n* = 44; 96.6%), with some topics covered in another subject (*n* = 3; 6.4%) or Science (*n* = 2; 4.3%). Lessons were mostly facilitated by a classroom teacher (*n* = 45; 95.7%), followed by an external facilitator (*n* = 20; 42.6%), someone else (*n* = 9; 19.1%), a school nurse (*n* = 2; 4.3%) or a school counsellor (*n* = 1; 2.1%).

Students reported their RSE to be ‘extremely relevant’ (*n* = 11; 23.9%) or ‘somewhat relevant’ (*n* = 21; 45.7%). There was no significant difference in perceived RSE relevance and gender (*χ* ^2^ = 8.630, df = 9, *p* = 0.472). Over 80% of students reported that 13 of the 18 topics had been covered in the RSE lessons. More students reported coverage of all topics compared to baseline (Table 4).

Most students reported ‘never’ feeling annoyed (74.5%; *n* = 35) or embarrassed (51.1%; *n* = 24) and about one‐third (36.2%; *n* = 17) reported ‘never’ feeling uncomfortable during RSE lessons. Approximately half (53.2%; *n* = 25) reported ‘sometimes’ feeling uncomfortable and 46.8% (*n* = 22) reported ‘sometimes’ feeling embarrassed during RSE lessons. There were no significant differences between feeling embarrassed (*χ* ^2^ = 5.172, df = 6, *p* = 0.522) or annoyed (*χ* ^2^ = 4.978, df = 6, *p* = 0.547) while girls were more likely to report feeling uncomfortable sometimes compared to males (*χ* ^2^ = 15.261, df = 6, *p* = 0.018).

### 3.2 School 1: Qualitative Findings

*RSE is systematically not prioritised*. Health was considered less of a priority compared to subjects like English and Maths: ‘literacy and numeracy are the priority … Health would come last on most people's priority list … I don't think the department encourages you to make it a priority’ (Talia, Teacher). Teachers suggested that often health lessons may be missed, particularly in times of heavy workloads; ‘if I … haven't got my English done … let's just let Health go…not talk about relationships this week … English is our core business … I need to focus on that’ (Briana, Teacher).

However, although considered a lower priority within the education system, participants personally felt RSE to be a priority: ‘you drop Health … what's that saying? We don't value it … that's rubbish … if kids don't know how to talk to each other … don't know how to be kind…and respectful …, then nothing else is going to work … to me it's priority number one’ (Layla, Teacher). It was recognised that other teachers may not be as passionate and committed and personal beliefs and choices may influence the depth and enthusiasm around including RSE. Prioritising RSE was not ‘the norm’, and would depend on the teacher, particularly, for those who may feel they need to catch up on content from other learning areas: ‘if they can stop teaching a subject to make way for their being behind in other subjects, the subject they will can is Health … it depends on who's teaching it as to … whether it's made a priority’ (Briana, Teacher).

### 3.3 Approaches to Age‐Appropriate Education

Teachers suggested all RSE topics could be delivered in a primary school setting in a developmentally appropriate manner. Participants discussed their experiences adapting RSE content for student readiness/age. For example, two teachers described tailoring lessons focusing on consent to be appropriate for the grade level: ‘things like consent … in a year six context … I don't teach them how to have sex … it is you know, respect your body, you've got the right to say no, if anybody touches you’ (Talia, Teacher) and ‘I do definitely in pre‐primary teach about consent … I'm a mum, do I have a right to just go and kiss my kids and hug them if they don't want that? And so they go ‘um yeah’, and ‘I go’ ‘no actually I don't, it's up to them’. So if grandma or grandpa wants a kiss, the child has the right to say ‘no’ … then we'd use hula hoops to show … our personal space … so it's taught at an age‐appropriate level’ (Brianna, Teacher). Similarly parents also discussed the importance of having open conversations in a developmentally appropriate manner, for example: ‘I'm a parent that tends to talk to my kids about anything and everything … they're going to go and see stuff on the internet that I would prefer, they would have spoken to me about first … I think it's important that you talk about a wide variety of things’ (Anne, Parent). Participants discussed that it was important to consider the age and developmental level of students, and to ensure the content, terminology and context of RSE topics meet their needs.

### 3.4 Schools Are Appropriate Environments for RSE Delivery

Parents discussed the benefit of including RSE within a school environment which may ‘take the burden off parents’ in having these kinds of conversations. For example, ‘I can't imagine I'd want to have a conversation with my children in regard to sexually transmitted diseases too often’, however, recognised that their child ‘knows … a lot about sex education … that's clearly come from school’ and ‘[they] don't have a problem with the school covering those topics’ (Brody, Parent). Belinda (Parent) suggested schools are appropriate environments as information about RSE coming from school staff is ‘better’, and the staff member delivering the information can make the students ‘feel more comfortable … in a safer environment.’

### 3.5 A Whole‐School Approach for Success

The support of school leadership was considered essential when implementing whole‐school RSE. Teachers noted the shift seen in the school due to participation in the intervention, ‘it's being supported now really well, from a school leadership and hierarchy perspective’ (Stephen, Teacher). Others noted that this support is evident through the leadership's ‘permission’ for them to prioritise this content. Talia said, ‘people have been given permission to spend the time … take half an hour out of your day to sit and talk with the kids, read them a book about relationships and then have a discussion … it gave people permission that this was just as valuable as doing a math worksheet’ (Talia, Teacher). Similarly, ‘because you've got leadership support, it gives us permission to do it … they're promoting what we're doing … it gives you permission to … stop and say, actually, this is important’ (Briana, Teacher). Furthermore, the inclusion of RSE within school board meetings, allowed parents and staff to discuss ‘about wellbeing and pastoral care’, while the previous focus was ‘about academics and what's the school doing for numeracy and literacy’ (Stephen, Teacher).

Parents discussed the pressure placed on teachers to deliver RSE and felt this should be a collaborative approach between parents and the school. Some parents felt that parents/carers should not rely solely on the school to teach RSE, ‘I think with all education … parents and teachers need to work together … I don't think everything should fall on the schools … parents should be taking part of that as well’ (Belinda, Parent). Adopting a collaborative approach to education, could provide students with *‘*a good starter’ (Anne, Parent) and reinforce the messages that are taught within the school environment. Anne recognised that schools are useful environments to provide information on RSE topics, and ‘they do try to make it a safe environment … they know that everyone around them is going through the same thing … it's a good thing to talk about it at school too’, however, parents cannot expect the school to address and teach everything, and ‘parents need to discuss it [RSE] with their kids at home as well’.

### 3.6 School 2: Quantitative Findings

Students from Grades 7–12 (*n* = 545) completed the pre‐intervention survey (Table 1). Most students (*n* = 416; 76.3%) reported receiving RSE at this school and had received RSE in Health (*n* = 471; 86.4%). RSE lessons were mostly facilitated by a classroom teacher (*n* = 410; 75.2%) or school nurse (*n* = 133; 24.4%) followed by an external facilitator (*n* = 103; 18.9%), school counsellor (*n* = 12; 2.2%) or chaplain (*n* = 12; 2.2%). Students reported their RSE to be somewhat relevant (*n* = 240; 48.98%); very relevant (*n* = 140; 28.57%) or extremely relevant (*n* = 47; 9.59%). There were no significant differences between the relevancy of RSE and grade level (*χ* ^2^ = 30.29, df = 20, *p* = 0.065) or RSE relevancy and gender (*χ* ^2^ = 10.508, df = 12, *p* = 0.571) (Table 5). Most students never felt embarrassed (*n* = 297; 62.79%) or annoyed (*n* = 314; 66.81%) during RSE, while 40.17% (*n* = 192) reported sometimes feeling uncomfortable. There were no significant differences between grade and feeling embarrassed (*χ* ^2^ = 9.149, df = 10, *p* = 0.518) or feeling annoyed (*χ* ^2^ = 13.302, df = 10, *p* = 0.207); however, younger students were significantly more likely to feel uncomfortable sometimes (*χ* ^2^ = 25.042, df = 10, *p* = 0.005). Females were slightly more likely to feel embarrassed (*χ* ^2^ = 15.727, df = 6, *p* = 0.015), uncomfortable (*χ* ^2^ = 41.147, df = 6, *p* = < 0.0001) and annoyed (*χ* ^2^ = 14.435, df = 6, *p* = 0.025) compared to males and other genders (Table 5).

**TABLE 5:** RSE relevancy for students and their feelings during RSE lessons (School 2).

	Grade 7	Grade 8	Grade 9	Grade 10	Grade 11	Grade 12	All grades	Male	Female	Other	Prefer not to answer
RSE relevancy											
Not relevant at all	16 (14.55)	8 (9.3)	9 (8.26)	8 (7.02)	0 (0)	1 (4.76)	42 (8.57)	20 (9)	2 (8.2)	2 (18.2)	0
Somewhat relevant	45 (40.91)	44 (51.16)	55 (50.46)	55 (48.25)	27 (52.94)	15 (71.43)	240 (48.98)	97 (43.9)	129 (52.9)	4 (36.4)	9 (75)
Very relevant	31 (28.18)	23 (26.74)	30 (27.52)	38 (33.33)	15 (29.41)	3 (14.29)	140 (28.57)	72 (32.6)	61 (25)	4 (36.4)	2 (16.7)
Extremely relevant	8 (7.27)	6 (6.98)	12 (11.01)	12 (10.53)	8 (15.69)	1 (4.76)	47 (9.59)	22 (10)	23 (9.4)	1 (9.1)	1 (8.3)
Not had RSE at school	10 (9.09)	5 (5.81)	3 (2.75)	1 (0.88)	1 (1.96)	1 (4.76)	21 (4.29)	10 (4.5)	11 (4.5)	0	0
*Feelings during RSE lessons*											
Embarrassed											
Always	7 (7)	8 (9.2)	7 (6.6)	6 (5.36)	2 (4.17)	2 (9.52)	32 (6.77)	10 (4.7)	20 (8.5)	1 (10)	1 (9.1)
Sometimes	31 (31)	34 (39.08)	34 (32.08)	29 (25.89)	11 (22.92)	5 (23.81)	144 (30.44)	53 (24.7)	87 (37)	1 (10)	2 (18.2)
Never	62 (62)	45 (51.72)	65 (6.32)	77 (68.75)	53 (72.92)	14 (66.67)	297 (62.79)	152 (70.7)	128 (54.5)	8 (80)	8 (72.7)
Total (*n*)	100	87	106	112	48	21	473	215	235	10	11
Uncomfortable											
Always	10 (9.8)	16 (18.18)	12 (11.21)	11 (9.82)	2 (4.08)	4 (19.05)	55 (11.51)	14 (6.6)	38 (15.7)	2 (20)	1 (9.1)
Sometimes	48 (47.06)	42 (47.73)	47 (43.93)	33 (29.46)	17 (34.69)	5 (23.81)	192 (40.17)	65 (30.5)	120 (49.6)	1 (10)	4 (36.4)
Never	44 (43.14)	30 (34.09)	48 (44.86)	68 (60.71)	30 (61.22)	12 (57.14)	231 (48.33)	134 (62.9)	84 (34.7)	7 (70)	6 (54.5)
Total (*n*)	102	88	107	112	49	21	478	213	242	10	11
Annoyed											
Always	8 (8)	5 (5.81)	12 (11.54)	5 (4.46)	1 (2.08)	1 (4.76)	32 (6.81)	11 (5.1)	19 (8.2)	1 (10)	1 (9.1)
Sometimes	21 (21)	18 (20.93)	25 (24.04)	37 (33.04)	16 (33.33)	7 (33.33)	124 (26.38)	41 (19.2)	76 (32.6)	3 (30)	2 (18.2)
Never	71 (71)	63 (73.26)	67 (64.42)	70 (62.5)	31 (64.58)	13 (61.9)	314 (66.81)	162 (75.7)	138 (59.2)	6 (60)	8 (72.7)
Total (*n*)	100	86	104	112	48	21	470	214	233	10	11

A variety of RSE topics were covered (Table 6). The most common topic across all year groups was puberty changes (*n* = 316; 58%). Over 40% of students indicated they had covered bullying, cyberbullying and the male and female reproductive systems. Less than 20% of students reported covering nine of the 23 topics, which included power in relationships (*n* = 97; 17.8%), sexual diversity and respect (*n* = 97; 17.8%), correct terminology of anatomy (*n* = 93; 17.1%), contraception (*n* = 89; 16.3%) and pornography (*n* = 68; 12.5%).

**TABLE 6:** Topics covered in School 2 at pre‐intervention.

Topics	Year level topic was covered						
	Grade 7	Grade 8	Grade 9	Grade 10	Grade 11	Grade 12	Total
Respect in relationships	24 (18.9)	24 (26.7)	69 (58)	56 (43.4)	2 (3.5)	8 (36.4)	183 (33.6)
Puberty changes	50 (39.4)	69 (76.7)	100 (84)	69 (53.5)	14 (26.4)	14 (63.6)	316 (58)
Female reproductive system	26 (20.5)	44 (48.9)	93 (78.2)	50 (38.8)	6 (10.5)	10 (45.5)	229 (42)
Male reproductive system	22 (17.3)	46 (51.1)	90 (75.6)	48 (37.2)	7 (12.3)	10 (45.5)	223 (40.9)
‘Our bodies are our own’	23 (18.1)	28 (31.1)	42 (35.3)	39 (30.2)	4 (7)	7 (31.8)	143 (26.2)
Correct terminology of anatomy	7 (5.5)	18 (20)	23 (19.3)	35 (27.1)	2 (3.5)	8 (36.4)	93 (17.1)
Sexually transmissible infections (STIs)	8 (6.3)	10 (11.1)	36 (30.3)	55 (42.6)	8 (14)	11 (50)	128 (23.5)
Blood‐borne viruses (BBVs)	3 (2.4)	6 (6.7)	10 (8.4)	18 (14)	1 (1.8)	6 (27.3)	44 (8.1)
HIV/AIDS	2 (1.6)	11 (12.2)	22 (18.5)	45 (34.9)	6 (10.5)	11 (50)	97 (17.8)
Sex and the law	16 (12.6)	17 (18.9)	50 (42)	45 (34.9)	4 (7)	7 (31.8)	139 (25.5)
Contraception	1 (0.8)	2 (2.2)	27 (22.7)	43 (33.3)	5 (8.8)	11 (50)	89 (16.3)
Power in relationships	8 (6.3)	9 (10)	39 (32.8)	34 (26.4)	2 (3.5)	5 (22.7)	97 (17.8)
Consent	5 (3.9)	4 (4.4)	45 (37.8)	56 (43.4)	18 (31.6)	10 (45.5)	138 (25.3)
Seeking sexual advice and information	14 (11)	12 (13.3)	43 (36.1)	44 (34.1)	3 (5.3)	11 (50)	127 (23.3)
Sexual diversity and respect	7 (5.5)	9 (10)	31 (26.1)	43 (33.3)	2 (3.5)	5 (22.7)	97 (17.8)
Cyber bullying	38 (29.9)	49 (54.4)	93 (78.2)	47 (36.4)	4 (7)	11 (50)	242 (44.4)
Bullying	52 (40.9)	53 (58.9)	96 (80.7)	47 (36.4)	6 (10.5)	11 (50)	265 (48.6)
Communication	16 (12.6)	23 (25.6)	41 (34.5)	39 (30.2)	3 (5.3)	7 (31.8)	129 (23.7)
Relationships	23 (18.1)	35 (38.9)	70 (58.8)	51 (39.5)	5 (8.8)	8 (36.4)	192 (35.2)
Love and intimacy	9 (7.1)	11 (12.2)	24 (20.2)	45 (34.9)	2 (3.5)	5 (22.7)	96 (17.6)
Gender identity	18 (14.2)	18 (20)	26 (21.8)	43 (33.3)	2 (3.5)	3 (13.6)	110 (20.2)
Pornography	6 (4.7)	10 (11.1)	23 (19.3)	23 (17.8)	2 (3.5)	4 (18.2)	68 (12.5)
Group rules	4 (3.1)	3 (3.3)	8 (6.7)	17 (13.2)	—	4 (18.2)	36 (6.6)
We have not covered any of these topics	26 (20.5)	4 (4.4)	3 (2.5)	6 (4.7)	1 (1.8)	—	40 (7.3)

### 3.7 School 2: Qualitative Findings

#### 3.7.1 Is RSE a Priority?

Teachers discussed broader system‐level prioritisation of more ‘academic’ subjects' impacted provision of RSE. Workload and competing academic priorities were highlighted as barriers. Participants noted RSE is mainly covered in Health, which is usually allocated one weekly lesson, and includes a wide variety of health issues. Consequently there is limited time to cover a comprehensive RSE curriculum and/or explore specific topics in detail, ‘one lesson a week is not enough, if we had two lessons a week, we'd be able to … put in all this extra stuff … there's just not enough hours in the week for us to teach and cover everything that's really important’ (Brooke, Teacher) and ‘we can't go into too much depth because we've got to hit all the points in the curriculum’ (Sandra, Teacher). Teachers suggested RSE is often seen as an ‘elective’, and mandating RSE would help ensure RSE was consistently prioritised: ‘the moment something is mandated, then everyone has that buy in … until then … they can just keep getting away with their outdated opinions’ (Matilda, Teacher).

A lack of teacher training around RSE impacted delivery. Molly (Teacher) noted RSE is seldom covered within typical university education, and therefore when teachers start delivering this content, they ‘find [teaching RSE] quite uncomfortable’. It was noted that teachers often lack the knowledge, skills and confidence to engage in discussions and deliver RSE content. Overall, teachers felt RSE was undervalued in the current system, not given enough time within the curriculum and many teachers are ill‐prepared to deliver contemporary content.

#### 3.7.2 Approaches to Age‐Appropriate Education

While teachers from this school were supportive of RSE they discussed the difficulty in introducing topics before students were mature enough to understand and critically engage with the content: ‘there's a right and wrong time … a lot of them are like, boys are too immature to take on board what you're saying’ (Brooke, Teacher). This sentiment was repeated by Matilda (Teacher), who reflected ‘I think it's really important, especially for our boys and our girls. There's conversations about you know the effect of porn … but God no, do not bring it up to my new sevens … maybe the last day of year 12 they might be mature enough’. Teachers discussed that although the content is important, there were some topics they did not feel comfortable including with younger students. Despite this, teachers recognised that not including some topics could be detrimental; for example, ‘to just kind of negate them or remove them from the curriculum almost gives them a chance to be more … taboo … if … we discussed them in an appropriate way, and explain the decisions that have been made around those sorts of topics, I think that it sort of removes the danger of it almost’ (Molly, Teacher). The importance of engaging in critical and open discussions about various RSE topics with students, while ensuring content is age and developmentally appropriate was reflected.

#### 3.7.3 Schools Are Appropriate Environments for RSE Delivery

There was a perceived need to provide factual and unbiased information, and participants felt some information from parents may be biassed and opinionated, ‘when they learn it [RSE] from home, it's all opinionated’ (Brooke, Teacher). When compared to the school environment, teachers noted that ‘you can't have an opinion in [RSE] … you just got to be factual’ (Sandra, Teacher). This was seen as beneficial, as school provides an opportunity for students to discuss RSE topics through a ‘very non‐biassed environment’ (Sandra, Teacher). Matilda noted the purpose of RSE lessons within school is not to change student opinions but to ‘try and teach them tolerance’ (Matilda, Teacher).

Teachers suggested parents can be uncomfortable discussing relationships and sexuality at home, and if they do receive information, it can be skewed by parental opinions or beliefs. Schools were reported as appropriate environments as the information is more likely to be factual and devoid of personal opinions and beliefs. Brooke (Teacher) said ‘we're not opinionated … when they learn it from home it's all opinionated … if we give the factual information … that gives them a little insight into … what it is’. Participants suggested students may be more comfortable discussing these topics with their teachers, especially when parents were uncomfortable with the content, ‘a lot of the students are more open with their teachers … than they are with their parents … it's not necessarily seen as something that's acceptable to talk about’ (Molly, Teacher).

This perception was also noted in the student FGD. Kelly (Student) stated ‘home relationships can be way different for every family … they teach things way differently’ and ‘if you leave it up to [home], kids might not get educated which means they don't learn.’ Another student reflected on teachings from their parents, suggesting ‘some parents just don't have the talk at all … I've never had the talk from my parents’ (Jim, student). Staff reinforced the sentiment that parents may not feel comfortable discussing elements of RSE or may not have the information and skills to discuss RSE topics; for example, ‘parents are rarely equipped with knowledge, because it's a fairly new thing for them, they're not able to give their children support’ (Mary, Teacher). Participants suggested schools play a role in ensuring young people receive consistent and factual information.

Students spoke about teachable moments, and how utilising these instances assists others in holding you accountable for your actions, For example: ‘if it's being taught around your peers, your peers can hold you to those standards … that's very important to do’ (Jim, Student) and ‘then you may know what's right or wrong … say … you just say something stupid, it's important for your peers to pull you up on it’ (Jim, Student). Students discussed that teaching RSE within a school environment enables the sharing of information amongst friendship groups and leaves students feeling accountable for addressing inappropriate behaviours and language.

#### 3.7.4 A Whole‐School Approach for Success

A whole‐school approach was seen as essential to ensure consistency of messages. Teachers noted the pressure they are placed under and the ‘blame’ they receive for students' behaviours and emphasised the need for message reinforcement in the home: ‘you see it in the media, every time there's something … there might be an issue about consent, ‘why aren't we teaching it in our schools’ it's like well … ‘why aren't you bloody‐well teaching it at home?’ You know … we do teach it at school, but it needs to be constantly reinforced*’* (Matilda, Teacher). Students also reinforced the need for a collaborative approach between home and school, noting this allows students to have someone safe they can approach about certain topics, depending on who they felt comfortable with; ‘I feel like it's good to get that talk at school and at home … sometimes you can't go to school about stuff and sometimes you can't go to your family about stuff. So, it's always good to have both choices’ (Sarah, Student).

Consistency of messages within the school was also needed, ‘I think it's essential that as a school and across the entire teaching staff, everyone shares the same message or has the same conceptual understanding of what's going on and how to discuss or approach those subjects with students’ (Molly, Teacher). Participants suggested a whole‐school approach should ensure that school staff have a mutual understanding of how to engage and respond to RSE related topics, ‘if kids are being homophobic in maths, and they're not being called out for it … it's not getting to … the year coordinator till [sic] lunchtime, it's too late … it should be at the forefront’ and ‘I think some of our teachers are probably not on board as much as others … which then becomes another problem, they sort of bring their own personal opinions into it … I know of teachers out on duty that have just kind of walked past it’ (Matilda, Teacher).

While Health teachers were seen as important facilitators of RSE, ‘those people [Health teachers] build better relationships with kids than what the average teacher does … they are able to broach across some more sensitive type topics with ease, and not feel uncomfortable about it’ (Michael, Teacher), all staff should be equipped with the skills, knowledge and confidence to teach and engage with RSE content. Teachers noted students form relationships with specific teachers, depending on whom they feel comfortable with, ‘just expecting … the Health teacher will cover it … means that students then have a limited number of people … if they don't feel comfortable with that limited number of people … they're not necessarily going to reach out when they need to’ (Molly, Teacher). Students also discussed the importance of student–teacher relationships ‘some kids don't get along with their Health teacher or feel uncomfortable with their Health teacher … it should be … I can talk to any teacher about that [RSE] … a teacher be just as able to answer the question that I have … it's important to be able to go talk to that person that you trust, rather than only having one source and that source may not be working for you’ (Kelly, Student). Support across the whole school was seen to be important, ‘if we need further support … they should be able to direct us to exactly here or here, having it so that if we're comfortable with a teacher, we can go to them’ (Ryan, Student). The need for school staff to receive training and support was discussed, emphasising the importance of support from the RSE Project: ‘if you have the speaker [RSE team member] come in and talk about it, the teachers are … better equipped with the knowledge to help out the students in situations if something's happened and they can't go home or talk about it … they'll have someone to talk about it with’ (Jim, Student).

The importance of embedding a culture of respect, understanding and acceptance within the whole‐school that extended from the Health curriculum was emphasised. The reinforcement of key messages and the creation of a safe environment, through modelling and use of teachable moments was discussed; for example ‘it becomes a little bit harder when you get to, say maths … but that doesn't mean that respectful relationships can't be modelled within the classroom, they may not necessarily be discussing … relationships and sexual education within the classroom, but they can acknowledge and be educating the students in how to engage with each other respectfully’ (Brooke, Teacher) and ‘even if … we're not teaching the nitty gritty of it … I mean in my classroom, we've got rainbow flags, we've got everything because it's just, I'm creating a safe environment … by doing that, I'm teaching consent, I'm teaching tolerance, I'm teaching respect’ (Brooke, Teacher).

Leadership support was seen to be essential for a whole‐school approach. Teachers reflected on changes since their involvement with the RSE Project: ‘I think … now there's a lot more buy in … before there was maybe a few teachers that had identified some issues … there wasn't really any buy in from the school’ and ‘the fact that [the Principal] went and got [the RSE Project] … it has been a real top‐down approach … I think if [the Principal] and other deputies were intolerant … it wouldn't have caught on’ (Brooke, Teacher). The significance of the school administration team supporting the programme, the RSE content and the whole‐school approach was highlighted, ‘while there was support mechanisms at school, I've gained great support from people like [RSE Project staff], who have helped push me in the direction that I needed to go to get support’ and having ‘someone to really push and hammer’ [RSE can really help] ‘get more of those things happening’ (Michael, Teacher).

## 4 Discussion

This study described case studies with schools implementing whole‐school RSE. Leadership support and staff commitment positively impacted the implementation of whole‐school RSE. With training and technical support schools were able to implement whole‐school RSE.

Survey data from the two schools highlighted the breadth of RSE topics covered with increases in the variety of topics taught over the years being notable. While most students found their RSE to be useful some reported ‘sometimes’ feeling embarrassed, uncomfortable and annoyed during RSE lessons. A New South Wales study exploring student (Grades 8–12) views and experiences of RSE, found some topics to be awkward and uncomfortable [46]. The way RSE is facilitated can impact students' experiences of RSE [24, 25, 47], especially when teachers feel awkward and uncomfortable with the content they are delivering [48]. New topics being covered may impact levels of student comfort [46]. There is a need for ongoing teacher training and support as new topics and issues emerge.

Despite the recognition that comprehensive RSE empowers young people through the enhancement of knowledge, skills and attitudes to positively impact their sexual health [1, 2, 3] and that various RSE topics are included in the WA Curriculum [19], some teachers in this study felt RSE was not given sufficient priority within the education system. Similarly, others have found crowded curriculum and teachers feeling unprepared or uncomfortable teaching RSE to be barriers to effective RSE [21, 23]. Similar to previous recommendations, greater emphasis on RSE in pre‐service [20, 39] and in‐service teacher training [40] is required. Teachers in this study were generally passionate about teaching RSE highlighting the importance of teacher preparedness and support to deliver this content.

Attitudes towards age‐appropriate RSE differed between the two‐case study schools. Participants from the primary school discussed the need for RSE topics and content to be tailored and altered to be delivered in an age‐appropriate way to all students, while secondary teachers were more likely to suggest that delivering age‐appropriate content would mean negating certain topics from lessons until students were ‘*mature enough*’ to understand [3] recommends that the content of RSE needs to be delivered in line with stages of emotional and cognitive development. However, they also propose ways in which all eight key concepts of comprehensive RSE can be taught across school‐ages. For example, topics such as healthy relationships, communication and consent content will vary in complexity and context based on age and should consider the individual school needs [3]. Age‐appropriate RSE has been identified as a human right for children [49] and RSE is considered most effective when delivered early, before the onset of sexual behaviour.

Parents in this study were supportive of introducing a wide variety of topics within their child's school‐based RSE. In contrast, one study found a lack of support from the broader school and parent community and parental concerns regarding topics covered in RSE can be barriers to implementing a comprehensive RSE programme [23]. However, an Australian study (*n* = 2427) found 89.9% of parents to be supportive of school‐based RSE. Over 80% of parents agreed that all 40 RSE listed topics should be covered within a school‐based RSE programme (topics included focus on peer relationships, sexual pleasure, gender identity and more) and of these parents 100% wanted these topics to be covered by Grade 8 [12]. There is a need to provide teaching staff with tailored support to deliver contemporary RSE topics in an age and developmentally appropriate manner.

Schools were seen to be ideal environments to facilitate whole‐school RSE by staff, parents and students as they provided a safe place for discussion and capacity for consistent messages. Training for all school staff, not only traditional teachers of RSE, was seen as especially salient given the opportunity for key RSE content to be delivered as a whole‐school approach [30] across the curriculum and within the school ethos and environment. The need for school leaders to advocate for and promote RSE was emphasised and evidenced by the two participating case study schools. The two schools involved in this evaluation had strong support from senior leadership and established effective working committees. To enhance sustainability and reduce burden existing committees can be leveraged. Schools that were lost to the study cited the loss of a school champion to advocate for the programme.

Case study schools implemented evidence‐based whole‐school strategies that were relevant to their individual school community. Technical support for school staff to support planning and implementation enhanced capacity.

This study faced several challenges due to the COVID‐19 pandemic. Usual challenges of finding time to conduct research in schools were exacerbated by school closures, restrictions and schools catching up on missed events. The team was unable to implement a post‐survey at the secondary school or to conduct FGDs with the primary school students or interviews with parents from the secondary school.

The cross‐sectional nature of this study does not enable programme impact to be determined. However, the purpose of this study was to understand implementation. The cross‐sectional data provided a good understanding of student attitudes and data collection presented minimal burden to schools.

The four case study schools involved in the broader project sought assistance from the RSE Project to improve their delivery of RSE within their school; hence, they were clearly committed. However, only two of these schools participated in the case study highlighting barriers to implementation despite initial enthusiasm.

The professional experiences, discipline perspectives and genders of the research team may have influenced data interpretation. The six phases of reflexive thematic analysis [44] have guided analysis and enabled robust discussion enabling the team to approach the data with awareness that our interpretations are shaped by these experiences and our genders.

## 5 Conclusion

The benefits of health promoting schools are well articulated; however, schools are often allocated limited resources and support to plan and implement strategies. This study recognised that while some staff are passionate about RSE, systemic restrictions may impact priorities, highlighting the importance of school leadership support. There is a need for support for school staff to deliver contemporary age and developmentally appropriate content that respects emotional and cognitive development and is inclusive of all genders, sexualities and diverse backgrounds. Consistency of messages within the school and home environments is needed to support whole‐school implementation. Findings emphasise the need for coordinated school staff training and support to ensure effective whole‐school RSE.

## Funding

This research was conducted as part of a broader contract with the Western Australian Department of Health (DoH20227687); Sexual Health and Blood‐borne Virus Program.

## Ethics Statement

This research was approved by the Curtin University Human Research Ethics Committee (HR91/2014) and the Department of Education Western Australia (D 18/0057006).

## Consent

Principals provided consent for school participation. All participants provided informed consent. Parental consent was also obtained for students.

## Conflicts of Interest

The authors declare no conflicts of interest.

## Data Availability

The data that support the findings of this study are available on request from the corresponding author. The data are not publicly available due to privacy or ethical restrictions.
